# Supplementary material for: Evolutionary radiation of earless frogs in the Andes: molecular phylogenetics and habitat shifts in high-elevation terrestrial breeding frogs
Source: PeerJ. 2018 Feb 22;6:e4313. doi: 10.7717/peerj.4313 (PMC5825883; doi:10.7717/peerj.4313)
Supplement: Supplemental Information 1 [file peerj-06-4313-s001.docx]

**Supplementary materials**

**Evolutionary radiation of earless frogs in the Andes: molecular phylogenetics and habitat shifts in high-elevation terrestrial breeding frogs**

Rudolf von May^1,2^, Edgar Lehr^3^, Daniel L. Rabosky^1^

**^1^** Museum of Zoology & Department of Ecology and Evolutionary Biology, University of Michigan, 2039 Ruthven Museums Building, 1109 Geddes Ave., Ann Arbor, MI 48109, USA

**^2^** Museum of Vertebrate Zoology, University of California, Berkeley, 3101 Valley Life Sciences Bldg., Berkeley, CA 94720, USA

**^3^** Department of Biology, Illinois Wesleyan University, P.O. Box 2900, Bloomington, IL 61701, USA

Corresponding author: Rudolf von May, rvonmay@umich.edu

**Raw data:** Data Archival locations

1) Morphological data: see **Table S1**

2) Molecular sequence data: see **Table S2** for GenBank accession numbers

3) Phylogenetic trees: see Supplementary Information 2

**Table S1.** Morphological data for 21 species of *Phrynopus* included in this study. Codes of museum collections are: MUSM = Museo de Historia Natural, Universidad Nacional Mayor de San Marcos, Lima, Peru; NMP6V = National Museum, Prague, Czech Republic; UMMZ = University of Michigan Museum of Zoology, Ann Arbor, USA. Field number code is: IWU = Illinois Wesleyan University, Bloomington, USA. The dataset included the following measurements: snout–vent length (SVL), tibia length (TL, distance from the knee to the distal end of the tibia), foot length (FL, distance from proximal margin of inner metatarsal tubercle to tip of Toe IV), head length (HL, from angle of jaw to tip of snout), head width (HW, at level of angle of jaw), horizontal eye diameter (ED), interorbital distance (IOD), upper eyelid width (EW), internarial distance (IND), eye–nostril distance (E-N, straight line distance between anterior corner of orbit and posterior margin of external nares).

| Museum Nbr. | Sex | Species | SVL | TL | FL | HL | HW | ED | IOD | EW | IND | E_N |
| --- | --- | --- | --- | --- | --- | --- | --- | --- | --- | --- | --- | --- |
| KU 291634 | F | *P. auriculatus* | 14.5 | 7.3 | 7.0 | 6.2 | 5.6 | 2.1 | 2.2 | 1.3 | 1.8 | 1.4 |
| KU 291633 | M | *P. auriculatus* | 13.4 | 6.9 | 6.8 | 6.0 | 5.2 | 2.1 | 2.0 | 1.3 | 1.6 | 1.4 |
| MUSM 31099 | F | *P. badius* | 19.1 | 7.1 | 7.9 | 6.4 | 6.5 | 2.1 | 2.3 | 1.4 | 1.9 | 1.5 |
| FMNH 282818 | F | *P. badius* | 21.0 | 7.5 | 8.5 | 7.0 | 7.6 | 2.3 | 2.4 | 1.4 | 1.9 | 1.7 |
| MTD 44342 | F | *P. barthlenae* | 26.1 | 8.5 | 10.6 | 8.1 | 9.4 | 2.7 | 3.6 | 1.9 | 1.9 | 2.4 |
| MHNSM 20603 | F | *P. barthlenae* | 28.1 | 8.8 | 11.2 | 8.6 | 10.0 | 2.7 | 3.0 | 1.9 | 1.8 | 2.1 |
| MTD 44341 | F | *P. barthlenae* | 30.2 | 10.1 | 12.5 | 9.4 | 10.8 | 3.1 | 3.1 | 2.2 | 2.1 | 2.5 |
| MTD 44343 | F | *P. barthlenae* | 30.4 | 9.3 | 11.9 | 9.3 | 10.8 | 3.2 | 3.3 | 2.2 | 2.0 | 2.4 |
| MHNSM 20606 | F | *P. barthlenae* | 35.0 | 10.8 | 13.1 | 9.6 | 12.6 | 3.4 | 4.2 | 2.5 | 2.4 | 2.7 |
| USNM 286919 | F | *P. bracki* | 19.8 | 7.4 | 7.1 | 7.3 | 7.8 | 2.4 | 2.3 | na | 2.2 | 1.5 |
| USNM 286918 | M | *P. bracki* | 15.7 | 6.2 | 5.2 | 5.8 | 6.2 | 2.1 | 1.9 | na | 2.0 | 1.3 |
| MHNJP 4400 | M | *P. bracki* | 16.2 | 6.2 | 5.8 | 5.8 | 6.0 | 1.9 | 1.8 | na | 2.0 | 1.3 |
| MTD 45072 | F | *P. bufoides* | 30.5 | 10.3 | 12.2 | 9.9 | 11.6 | 3.5 | 3.7 | 1.9 | 2.6 | 2.2 |
| MTD 45074 | F | *P. bufoides* | 32.0 | 11.1 | 11.5 | 10.6 | 10.8 | 3.1 | 3.6 | 2.4 | 2.5 | 2.2 |
| MHNSM 18074 | F | *P. bufoides* | 32.9 | 11.6 | 13.6 | 10.6 | 11.4 | 3.4 | 4.1 | 2.2 | 2.4 | 2.4 |
| MUSA 4916 | F | *P. daemon* | 21.4 | 7.8 | 8.0 | 7.0 | 8.0 | 1.6 | 2.6 | 2.0 | 2.1 | 1.6 |
| CORBIDI 15364 | F | *P. daemon* | 24.4 | 8.2 | 9.6 | 7.9 | 8.1 | 1.8 | 2.6 | 2.0 | 2.1 | 1.8 |
| MUSM 32747 | M | *P. daemon* | 21.7 | 8.5 | 9.0 | 7.8 | 7.6 | 2.3 | 2.6 | 1.7 | 1.9 | 1.8 |
| SMF 80472 | F | *P. heimorum* | 20.0 | 6.5 | 7.6 | 5.6 | 6.2 | 1.9 | 1.7 | 1.7 | 1.5 | 1.3 |
| MHNSM 20443 | F | *P. heimorum* | 22.0 | 7.5 | 8.2 | 5.7 | 7.7 | 1.8 | 2.8 | 1.7 | 1.9 | 1.7 |
| MHNSM 20441 | F | *P. heimorum* | 23.5 | 7.8 | 8.6 | 5.7 | 7.6 | 2.0 | 2.6 | 1.5 | 1.7 | 1.8 |
| SMF 80470 | F | *P. heimorum* | 24.0 | 7.4 | 8.6 | 6.2 | 7.2 | 1.9 | 2.4 | 1.5 | 1.7 | 1.8 |
| MHNSM 20440 | F | *P. heimorum* | 24.4 | 7.1 | 8.4 | 6.1 | 7.5 | 1.7 | 2.3 | 1.8 | 1.9 | 1.9 |
| SMF 80474 | F | *P. heimorum* | 24.6 | 7.9 | 8.5 | 6.6 | 7.2 | 1.9 | 2.2 | 1.7 | 1.7 | 2.0 |
| SMF 80469 | F | *P. heimorum* | 24.9 | 7.3 | 8.5 | 6.1 | 7.8 | 2.1 | 2.5 | 1.6 | 2.0 | 1.5 |
| MHNSM 20442 | F | *P. heimorum* | 25.3 | 7.3 | 8.1 | 6.5 | 7.5 | 1.9 | 2.4 | 1.7 | 2.0 | 1.5 |
| MHNSM 20445 | F | *P. heimorum* | 25.5 | 7.5 | 8.0 | 6.3 | 7.3 | 2.0 | 2.2 | 1.5 | 1.8 | 1.7 |
| SMF 80471 | F | *P. heimorum* | 26.0 | 7.5 | 8.1 | 6.1 | 7.6 | 1.8 | 2.6 | 1.6 | 2.0 | 1.7 |
| MHNSM 20424 | F | *P. horstpauli* | 30.8 | 12.2 | 12.7 | 9.3 | 10.9 | 2.6 | 3.3 | 2.0 | 2.5 | 2.5 |
| MHNSM 20434 | F | *P. horstpauli* | 31.9 | 11.8 | 13.5 | 9.9 | 11.3 | 3.1 | 3.0 | 2.0 | 2.3 | 2.4 |
| SMF 80453 | F | *P. horstpauli* | 32.7 | 12.5 | 14.0 | 9.9 | 12.1 | 3.6 | 3.5 | 2.5 | 3.0 | 2.7 |
| SMF 80457 | F | *P. horstpauli* | 33.4 | 11.9 | 13.0 | 10.4 | 12.3 | 3.7 | 3.4 | 2.3 | 3.1 | 3.0 |
| MHNSM 20435 | F | *P. horstpauli* | 33.6 | 12.3 | 14.0 | 8.8 | 11.3 | 3.2 | 2.9 | 2.4 | 2.7 | 2.6 |
| MHNSM 20436 | F | *P. horstpauli* | 35.2 | 12.1 | 13.8 | 9.3 | 11.9 | 3.4 | 3.5 | 2.3 | 2.7 | 2.9 |
| SMF 80454 | F | *P. horstpauli* | 35.9 | 12.6 | 14.2 | 10.1 | 12.4 | 3.6 | 3.3 | 2.8 | 3.3 | 3.1 |
| SMF 80464 | F | *P. horstpauli* | 37.1 | 12.7 | 14.6 | 10.2 | 12.3 | 3.2 | 3.5 | 2.3 | 2.8 | 2.9 |
| MHNSM 20437 | F | *P. horstpauli* | 39.7 | 13.0 | 15.3 | 9.9 | 12.3 | 3.5 | 4.3 | 2.5 | 3.2 | 3.0 |
| SMF 80458 | M | *P. horstpauli* | 25.6 | 9.3 | 9.7 | 7.8 | 8.8 | 2.8 | 2.6 | 2.3 | 1.9 | 1.8 |
| MUSM 29543 | F | *P. interstinctus* | 23.8 | 9.8 | 10.2 | 7.7 | 9.2 | 1.9 | 2.2 | 1.9 | 2.3 | 2.0 |
| MUSM 29544 | M | *P. interstinctus* | 14.9 | 7.0 | 6.7 | 5.6 | 5.6 | 1.8 | na | na | 1.9 | 1.5 |
| MUSM 29545 | M | *P. interstinctus* | 16.5 | 7.6 | 7.4 | 6.1 | 5.8 | 2.1 | 2.0 | 1.3 | 1.9 | 1.4 |
| MUSM 31968 | F | *P. inti* | 40.4 | 15.7 | 17.1 | 13.5 | 14.5 | 3.5 | 3.5 | 3.4 | 3.1 | 3.0 |
| MUSM 31203 | M | *P. inti* | 27.2 | 10.3 | 12.2 | 9.4 | 10.5 | 2.4 | 3.0 | 2.5 | 2.1 | 1.9 |
| UMMZ 245220 | M | *P. inti* | 27.4 | 9.9 | 11.6 | 9.9 | 10.6 | 2.7 | 2.8 | 2.4 | 2.5 | 1.9 |
| MUSM 31183 | M | *P. inti* | 32.5 | 12.7 | 14.0 | 11.3 | 12.5 | 3.4 | 3.5 | 3.3 | 2.9 | 2.3 |
| NMP6V 75584 | M | *P. inti* | 34.2 | 14.0 | 15.4 | 11.4 | 12.2 | 3.1 | 3.7 | 3.2 | 2.7 | 2.1 |
| MUSM 31984 | M | *P. inti* | 35.1 | 13.3 | 13.7 | 11.9 | 12.6 | 3.4 | 3.0 | 2.8 | 2.6 | 2.5 |
| MUSM 31976 | M | *P. inti* | 35.2 | 14.0 | 13.8 | 13.0 | 13.4 | 3.1 | 3.7 | 3.3 | 2.9 | 2.6 |
| MUSM 33258 | F | *P. juninensis* | 33.0 | 10.6 | 11.9 | 10.8 | 10.5 | 3.0 | 2.8 | 1.9 | 2.7 | 2.5 |
| RvM57_14 | M | *P. juninensis* | 21.1 | 8.1 | 9.1 | 7.5 | 7.5 | 2.4 | 2.5 | 1.8 | 1.9 | 2.1 |
| RvM55_14 | M | *P. juninensis* | 22.2 | 8.9 | 9.1 | 7.8 | 7.7 | 2.3 | 2.5 | 1.9 | 1.8 | 2.0 |
| MHNSM 20459 | F | *P. kauneorum* | 29.1 | 11.5 | 13.0 | 9.6 | 11.2 | 3.1 | 2.8 | 2.8 | 2.5 | 2.9 |
| SMF 80626 | F | *P. kauneorum* | 32.2 | 12.9 | 14.0 | 9.1 | 12.7 | 3.0 | 3.4 | 2.8 | 2.9 | 3.1 |
| MHNC 6469 | F | *P. miroslawae* | 29.2 | 10.2 | 11.4 | 9.7 | 11.4 | 2.8 | 4.4 | 2.3 | 2.5 | 1.9 |
| MUSM 33260 | F | *P. montium* | 20.9 | 8.1 | 8.5 | 7.1 | 7.2 | 2.3 | 2.0 | 1.4 | 1.8 | 1.4 |
| MUSM 33259 | F | *P. montium* | 26.3 | 10.3 | 10.7 | 8.3 | 8.9 | 2.7 | 2.6 | 1.5 | 2.4 | 2.0 |
| UMMZ 89477 A | F | *P. peruanus* | 23.6 | 8.5 | 8.4 | 8.6 | 8.0 | 2.2 | 2.6 | 1.5 | 1.9 | 1.6 |
| MHNSM 19977 | F | *P. peruanus* | 27.5 | 9.7 | 9.3 | 8.8 | 9.8 | 2.5 | 2.8 | 1.9 | 2.0 | 1.0 |
| MTD 46801 | F | *P. peruanus* | 28.2 | 10.7 | 9.4 | 9.7 | 10.8 | 2.6 | 2.9 | 2.0 | 2.2 | 2.0 |
| MHNSM 19978 | M | *P. peruanus* | 22.1 | 7.8 | 7.4 | 7.7 | 8.4 | 1.9 | 2.4 | 1.5 | 1.7 | 1.6 |
| MTD 46802 | M | *P. peruanus* | 22.5 | 8.0 | 7.9 | 7.9 | 8.5 | 2.1 | 2.5 | 1.7 | 1.8 | 1.7 |
| UMMZ 89477 B | M | *P. peruanus* | 23.7 | 8.3 | 9.1 | 9.0 | 8.9 | 2.0 | 2.5 | 1.2 | 2.0 | 1.6 |
| NA_average | F | *P. pesantesi* | 29.1 | 10.3 | 10.3 | 9.2 | 10.0 | 2.9 | 3.3 | 2.4 | 2.1 | 1.8 |
| NA_average | M | *P. pesantesi* | 22.6 | 7.7 | 8.3 | 7.6 | 8.2 | 2.2 | 2.7 | 1.9 | 1.8 | 1.6 |
| MHNSM 19857 | M | *P. pesantesi* | 25.6 | 8.6 | 9.4 | 8.0 | 9.2 | 2.7 | 3.0 | 2.0 | 1.9 | 1.3 |
| RvM61_14 | F | *P. spI* | 25.6 | 10.4 | 11.3 | 9.0 | 9.3 | 2.9 | 3.0 | 2.0 | 2.3 | 2.0 |
| RvM59_14 | F | *P. spI* | 25.8 | 9.8 | 10.2 | 8.3 | 9.0 | 2.7 | 2.8 | 2.0 | 2.3 | 2.0 |
| RvM60_14 | M | *P. spI* | 18.8 | 7.8 | 8.0 | 7.3 | 7.5 | 2.2 | 2.5 | 2.3 | 1.8 | 1.4 |
| MHNSM 20612 | F | *P. tautzorum* | 28.9 | 8.9 | 10.8 | 8.1 | 10.1 | 3.1 | 3.2 | 2.2 | 2.3 | 1.8 |
| MTD 44348 | F | *P. tautzorum* | 29.9 | 9.1 | 10.8 | 9.0 | 9.9 | 3.2 | 3.2 | 2.1 | 2.4 | 2.0 |
| MUSM 31106 | F | *P. tribulosus* | 20.7 | 10.2 | 10.3 | 6.9 | 7.0 | 2.4 | 2.3 | 1.9 | 2.1 | 1.8 |
| MHNC 6441 | F | *P. tribulosus* | 21.2 | 9.0 | 10.1 | 6.1 | 6.9 | 2.3 | 3.0 | 1.2 | 2.1 | 1.8 |
| KU 291630 | M | *P. tribulosus* | 15.2 | 6.7 | 6.8 | 5.3 | 5.1 | 2.0 | 1.7 | 1.1 | 1.7 | 1.3 |
| MUSM 32748 | F | *P. unchog* | 26.9 | 8.9 | 9.9 | 9.3 | 8.5 | 2.3 | 2.6 | 2.0 | 2.1 | 2.0 |
| MUSM 32749 | M | *P. unchog* | 20.4 | 8.3 | 8.4 | 7.6 | 7.0 | 1.9 | 2.3 | 1.8 | 1.7 | 1.6 |
| MUSM 29542 | F | *P. vestigiatus* | 18.8 | 7.9 | 8.8 | 6.7 | 6.6 | 1.6 | 2.3 | 1.1 | 1.9 | 1.7 |

**Table S2.** GenBank accession numbers for the taxa and genes sampled in this study. Bold font indicates new sequences generated for this study. Taxonomy follows Padial et al. (2014).

| **Taxon** | **16S** | **12S** | **COI** | **RAG1** | **Tyr** | **Voucher_Nbr** |
| --- | --- | --- | --- | --- | --- | --- |
| *Hypodactylus brunneus* | EF493357 | EF493357 | na | EF493422 | EF493484 | KU178258 |
| *Hypodactylus dolops* | EF493394 | EF493394 | na | EF493414 | EF493483 | na |
| *Ischnocnema guentheri* | EF493533 | EF493533 | na | na | EF493510 | na |
| *Lynchius flavomaculatus* | EU186667 | EU186667 | na | EU186745 | EU186766 | KU218210 |
| *Lynchius nebulanastes* | EU186704 | EU186704 | na | na | na | KU181408 |
| *Lynchius oblitus* | AM039639 | AM039707 | na | na | na | MTD45954 |
| *Lynchius oblitus* | AM039640 | AM039708 | na | na | na | MHSNM19914 |
| *Lynchius oblitus* | KX470782 | KX470775 | na | KX470791 | na | MHNC8652 |
| *Lynchius oblitus* | KX470783 | KX470776 | na | KX470792 | KX470799 | MHNC8614 |
| *Lynchius oblitus* | KX470784 | KX470777 | na | na | KX470800 | MHNC8674 |
| *Lynchius parkeri* | EU186705 | EU186705 | na | na | na | KU181307 |
| *Lynchius simmonsi* | JF810004 | JF809940 | na | JF809915 | JF809894 | QZ41639 |
| *Lynchius tabaconas* | KX470780 | KX470773 | na | na | KX470796 | MHNC8637 |
| *Lynchius tabaconas* | KX470781 | KX470774 | na | na | KX470797 | MHNC8650 |
| *Oreobates amarakaeri* | JF809996 | JF809934 | na | JF809913 | JF809891 | MHNC6975 |
| *Oreobates ayacucho* | JF809970 | JF809933 | na | JF809912 | JF809890 | MNCN_IDlR5024 |
| *Oreobates cruralis* | EU186666 | EU186666 | na | EU186743 | EU186764 | KU215462 |
| *Oreobates gemcare* | JF809960 | JF809930 | na | JF809909 | na | MHNC6687 |
| *Oreobates granulosus* | EU368897 | JF809929 | na | JF809908 | JF809887 | MHNC3396 |
| *Phrynopus auriculatus* | EF493708 | EF493708 | na | na | na | KU291634 |
| *Phrynopus auriculatus* | MF186348 | MF186290 | MF186466 | na | MF186582 | MUBI 6471 |
| *Phrynopus badius* | **MG896571** | **MG896594** | **MG896611** | **MG896618** | na | FMNH282818 |
| *Phrynopus badius* | **MG896572** | **MG896595** | **MG896612** | **MG896619** | na | MUSM31099 |
| *Phrynopus barthlenae* | AM039653 | AM039721 | na | na | na | SMF81720 |
| *Phrynopus barthlenae* | MF186350 | MF186292 | MF186464 | na | na | MHNSM20609 |
| *Phrynopus bracki* | EF493709 | EF493709 | na | EF493421 | na | USNM286919 |
| *Phrynopus bufoides* | AM039645 | AM039713 | na | na | na | MHNSM19860 |
| *Phrynopus curator* | **MG896573** | **MG896596** | **MG896613** | **MG896620** | na | MUSM31106 |
| *Phrynopus daemon* | **MG896574** | **MG896597** | na | na | na | MUSM32747 |
| *Phrynopus heimorum* | AM039635 | AM039703 | MF186462 | MF186545 | MF186580 | MTD45621 |
| *Phrynopus heimorum* | AM039636 | AM039704 | na | na | na | MTD45622 |
| *Phrynopus horstpauli* | AM039647 | AM039715 | na | na | na | MTD44334 |
| *Phrynopus horstpauli* | AM039651 | AM039719 | na | na | na | MTD44333 |
| *Phrynopus horstpauli* | MF186364 | MF186303 | na | na | MF186584 | MTD44335 |
| *Phrynopus interstinctus* | **MG896575** | **MG896598** | **MG896614** | **MG896621** | na | MUSM29543 |
| *Phrynopus inti* | MF651901 | na | na | MF651916 | na | MUSM31203 |
| *Phrynopus inti* | MF651902 | MF651909 | na | MF651917 | na | MUSM31968 |
| *Phrynopus inti* | MF651903 | MF651910 | na | na | na | MUSM31976 |
| *Phrynopus inti* | MF651904 | MF651911 | na | na | na | MUSM31984 |
| *Phrynopus inti* | MF651905 | MF651912 | na | na | na | NMP6V75584 |
| *Phrynopus inti* | MF651906 | MF651913 | na | MF651918 | MF651921 | UMMZ_245218 |
| *Phrynopus inti* | MF651907 | MF651914 | na | MF651919 | na | UMMZ_245219 |
| *Phrynopus juninensis* | **MG896576** | **MG896599** | na | **MG896622** | na | MUSM38323 |
| *Phrynopus juninensis* | **MG896577** | **MG896600** | na | **MG896623** | na | MUSM38324 |
| *Phrynopus juninensis* | **MF651908** | **MF651915** | na | **MF651920** | na | MUSM33258 |
| *Phrynopus kauneorum* | AM039650 | AM039718 | na | na | na | MTD44332 |
| *Phrynopus kauneorum* | AM039655 | AM039723 | na | na | na | MHNSM20595 |
| *Phrynopus miroslawae* | MF186393 | MF186312 | MF186463 | MF186542 | MF186585 | MUBI 6469 |
| *Phrynopus montium* | **MG896578** | **MG896601** | na | **MG896624** | na | MUSM33259 |
| *Phrynopus montium* | **MG896579** | **MG896602** | na | **MG896625** | na | MUSM33260 |
| *Phrynopus nicoleae* | MF186394 | MF186313 | MF186468 | MF186546 | MF186577 | MUBI 6441 |
| *Phrynopus peruanus* | **MG896581** | **MG896604** | na | na | na | MUSM38315 |
| *Phrynopus peruanus* | **MG896582** | **MG896605** | **MG896615** | **MG896626** | **MG896631** | MUSM38316 |
| *Phrynopus peruanus* | **MG896583** | na | na | **MG896627** | **MG896632** | MUSM38317 |
| *Phrynopus peruanus* | **MG896584** | na | na | **MG896628** | na | MUSM38318 |
| *Phrynopus peruanus* | **MG896585** | na | na | na | na | MUSM38319 |
| *Phrynopus peruanus* | **MG896586** | na | **MG896616** | na | na | MUSM38320 |
| *Phrynopus peruanus* | **MG896587** | na | na | na | na | MUSM38321 |
| *Phrynopus peruanus* | **MG896588** | na | na | na | na | MUSM38322 |
| *Phrynopus peruanus* | **MG896580** | **MG896603** | na | na | na | MTD46801 |
| *Phrynopus pesantesi* | AM039656 | AM039724 | na | na | na | MTD45072 |
| *Phrynopus* sp | AM039657 | AM039725 | na | na | na | MTD45075 |
| *Phrynopus* sp | AM039660 | AM039728 | na | na | na | MTD44759 |
| *Phrynopus* spI | **MG896589** | **MG896606** | na | **MG896629** | na | MUSM33261 |
| *Phrynopus* spI | **MG896590** | **MG896607** | na | **MG896630** | na | MUSM33262 |
| *Phrynopus tautzorum* | AM039652 | AM039720 | na | na | na | MHNSM20613 |
| *Phrynopus tribulosus* | EU186725 | EU186707 | na | na | na | KU291630 |
| *Phrynopus tribulosus* | MF186423 | MF186329 | MF186469 | na | MF186578 | MUBI 6451 |
| *Phrynopus tribulosus* | MF186424 | MF186330 | MF186467 | MF186547 | MF186579 | MUBI 7166 |
| *Phrynopus unchog* | **MG896591** | **MG896608** | na | na | na | MUSM32748 |
| *Phrynopus unchog* | **MG896592** | **MG896609** | na | na | na | MUSM32749 |
| *Phrynopus vestigiatus* | **MG896593** | **MG896610** | **MG896617** | na | na | MUSM29542 |

**Table S3.** Primers used in this study.

| **Locus** | **Primer** |  | **Sequence (5'-3')** | **Reference** |
| --- | --- | --- | --- | --- |
| 16S | 16SAR | F | CGCCTGTTTATCAAAAACAT | Palumbi et al. (1991) |
|  | 16SBR | R | CCGGTCTGAACTCAGATCACGT | Palumbi et al. (1991) |
| 12S | L25195 | F | AAACTGGGATTAGATACCCCACTA | Palumbi et al. 1991 |
|  | H2916 | R | GAGGGTGACGGGCGGTGTGT | Palumbi et al. 1991 |
| COI | dgLCO1490 | F | GGTCAACAAATCATAAAGAYATYGG | Meyer et al. (2005) |
|  | dgHCO2198 | R | TAAACTTCAGGGT GACCAAARAAYCA | Meyer et al. (2005) |
| RAG1 | R182 | F | GCCATAACTGCTGGAGCATYAT | Heinicke et al. (2007) |
|  | R270 | R | AGYAGATGTTGCCTGGGTCTTC | Heinicke et al. (2007) |
| Tyr | Tyr1C | F | GGCAGAGGAWCRTGCCAAGATGT | Bossuyt and Milinkovitch (2000) |
|  | Tyr1G | R | TGCTGGGCRTCTCTCCARTCCCA | Bossuyt and Milinkovitch (2000) |

**Figure S1.** Pairwise scatterplot matrix displaying the correlation between relevant pairs of variables measured in this study (SVL, body-size corrected morphological data, and elevation); Pearson correlation values (font size scaled to value) are displayed on the lower half of the matrix. Abbreviations for each variable are included along the diagonal (Elev_Midpoint = elevational midpoint; SVL = snout-vent-length; TL = tibia length, FL = foot length, HL = head length, IOD = interorbital distance, IND = internarial distance, E_N = eye–nostril distance). Notice the relationship between elevational midpoint and other variables (top row). Some variables (HW = head width, ED = eye diameter, EW = eyelid width) with Pearson correlation values <0.55 (i.e., R^2^ values <0.30) were not included.
